# Molecular Imprinting Techniques Used for the Preparation of Biosensors

**DOI:** 10.3390/s17020288

**Published:** 2017-02-04

**Authors:** Gizem Ertürk, Bo Mattiasson

**Affiliations:** 1 CapSenze Biosystems AB, Lund SE-22363, Sweden; bo.mattiasson@biotek.lu.se; 2 Department of Biotechnology, Lund University, Lund SE-22369, Sweden

**Keywords:** molecular imprinting, biomimetic sensors, bulk imprinting, surface imprinting, epitope imprinting

## Abstract

Molecular imprinting is the technology of creating artificial recognition sites in polymeric matrices which are complementary to the template in their size, shape and spatial arrangement of the functional groups. Molecularly imprinted polymers (MIPs) and their incorporation with various transducer platforms are among the most promising approaches for detection of several analytes. There are a variety of molecular imprinting techniques used for the preparation of biomimetic sensors including bulk imprinting, surface imprinting (soft lithography, template immobilization, grafting, emulsion polymerization) and epitope imprinting. This chapter presents an overview of all of these techniques with examples from particular publications.

## 1. Introduction

Molecular imprinting is the technology of creating artificial recognition sites in polymeric matrices which are complementary to the template in their size, shape and spatial arrangement of the functional groups. Recently, molecular imprinting technology has been used for the creation of biorecognition surfaces on biosensors. There are different types of molecular imprinting techniques, including bulk imprinting, surface imprinting and epitope imprinting.

The template molecule is imprinted as a whole in the polymer matrix in bulk imprinting, whereas only a small part or a fragment of a macromolecule is imprinted as a representative of the whole molecule in epitope imprinting. Surface imprinting is a popular and convenient technique, especially for biomacromolecules including proteins. There are different types of surface imprinting like soft lithography, template immobilization including microcontact imprinting, grafting and emulsion polymerization which have been used successfully in previous reports. All of these techniques have some advantages and at the same time limitations when it comes into applications, however, in general; molecular imprinting technology is very promising to create highly specific, sensitive and long-term stable biorecognition cavities on biosensor surfaces to be used in many applications.

## 2. Molecular Imprinting Techniques

Molecularly imprinted polymers (MIPs) are prepared by co-polymerization of cross-linking monomer and a complex which is pre-formed between the template molecule and functional monomers using covalent, non-covalent or semi-covalent interactions. When the template molecule is removed from the imprinted material after polymerization, it leaves behind specific cavities that are complementary to the template in size, shape and chemical functionality [1]. MIPs have been used successfully in many applications, including purification [2], isolation [3], chiral separation [4], catalysis [5] and in biosensors [6,7]. For the imprinting of molecules on biosensor surfaces, there are different types of molecular imprinting techniques that can be classified in three main categories; bulk imprinting, surface imprinting and epitope imprinting.

### 2.1. Bulk Imprinting

In bulk imprinting, a template molecule is imprinted as a whole in the polymer matrix and after polymerization it needs to be wholly removed from the molecularly imprinted material. In the next step, to form small particles from these bulk polymers, the bulk polymer is crushed mechanically and the formed particles are fractionated. By this way, readily accessible, template-specific 3D interaction sites are obtained within the selective, imprinted polymeric material [8,9,10]. Bulk imprinting is generally preferred for the imprinting of small molecules since, at least in theory, the adsorption and release of the template molecule are faster and reversible which is an advantage for the re-usability of the imprinted support for several rounds of analyses [9].

The use of whole proteins as a template presents some advantages over other methods especially in sensor applications [11,12]. The template structure will most accurately be the same with the target structure since the template protein (the target, at the same time) is imprinted wholly. However, in case of larger structures including proteins, living cells and microorganisms, the method has some drawbacks. Maintaining the conformational stability of a protein during the polymerization process is a big challenge [13]. Moreover, when large imprinted sites are formed due to the size of the template, these sites might also be attractive for smaller polypeptides which results in cross-reactivity and reduced selectivity [13]. Due to the thick morphology of bulk imprints, big template molecules are embedded in the matrices too deeply that lead to restriction or no access for the target molecules to bind to the sites. The fact of low accessibility leads to much longer response times, drift problems and also poor regeneration [9,14]. A more extreme position is that the target molecule is problematic to remove from the MIP. This will lead to hampered binding and in extreme cases to no binding at all. To overcome these limitations, alternative imprinting techniques including surface and epitope imprinting have been developed.

### 2.2. Surface Imprinting

High affinity recognition sites are formed at the surface of a substrate in surface imprinting [15]. Due to this the recognition sites are more easily accessible with favorable binding kinetics. In other words, template/polymer interactions are not diffusion limited to the same extent as was a general problem encountered in bulk imprinting [16,17,18]. Therefore, the technique is popular and most applicable especially for imprinting of biomolecules including proteins. In surface imprinting, less template molecules are used as compared to what is used in conventional imprinting techniques because template is only used in the surface coating step in the technique [14]. The main drawback of the method in sensor design is the possibility of lower sensitivity as compared to bulk imprinting owing to the reduced number of imprinted sites [9]. Surface imprinted polymers have been widely used for different types of analytes, including proteins [19,20], microorganisms and cells [10,21].

#### 2.2.1. Soft Lithography

Soft lithography or stamping is one of the techniques that has been used for the preparation of (biomimetic) sensors by surface imprinting [22,23]. Micro and nano-scaled patterns can be prepared by this technique without any need for expensive and specialized equipment [9]. The technique was first introduced by Bain and Whitesides in 1989 [24]. In the general procedure of the method, micro- or nano-scaled patterns ranging from 30 nm to 100 µm are formed on the solid substrates by using a soft polymeric stamp [23]. A pre-polymerized layer is coated on a transducer surface and the template stamp is pressed over this surface for a certain period of time. Self-assembling of template structures including proteins, microorganisms onto a smooth support are used to produce the template stamps which are complementary to the template in their structural, geometrical and also chemical features. At the end of the procedure, highly selective and specific surface imprinted films are obtained via this technique.

For the preparation of stamp, one of the important points that has to be considered is the amount of template on the stamp which has a strong influence in sensor response. Dickert et al. [25] reported that multi-layer stamps showed better sensor response than mono-layer covered polymers. For the selection of solid support, a glass slide can be preferred owing to its rigid structure which means mechanical stability [26]. A relatively flexible material poly (dimethyl siloxane) is also used for more fragile structured templates [27]. Furthermore, polymer’s composition, polarity and methodology are also important parameters to obtain the optimal recognition between the template and the imprinted cavities [9,28]. After template removal process, it is important for the stability of the sensor surface to retain the shape of the imprinted cavities, which means the stability of the sensor surface is important for the binding efficiency.

**Figure 1:**
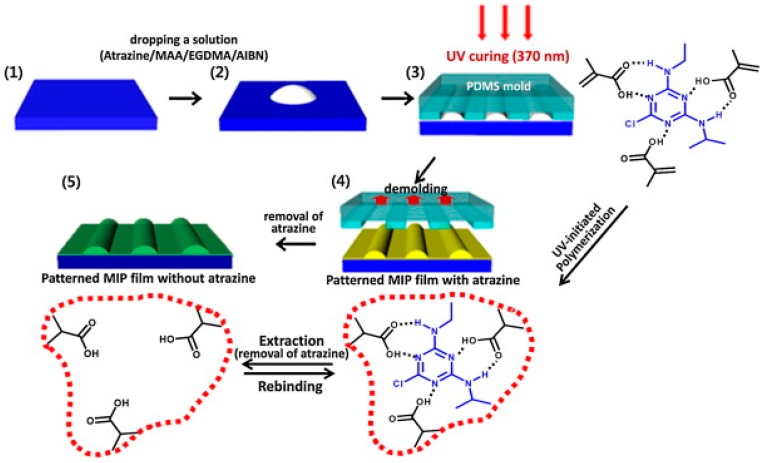
Schematic representation of fabrication process of patterned MIP films via soft lithography and UV polymerization. (**1**–**2**) Imprinting solution containing the template molecule, functional and cross-linking monomers and initiator was dropped on the substrate. (**3**) The patterned PDMS mold was placed on the droplet of solution under certain pressure to ensure enough physical contact between the PDMS and substrate. Then, UV polymerization was initiated and continued for 7–10 min. (**4**) Following de-molding, the patterned MIP films were dried at 60 °C. (**5**) In the last step, template molecule (atrazine) was removed from the surface (reproduced from [29] with permission).

A solvent-assisted soft lithography and UV-initiated polymerization were used to develop striped poly(methacrylic acid-ethylene glycol dimethacrylate) patterns for detection of traces of atrazine in aqueous solutions by using gravimetric QCM (Figure 1). This patterned MIP matrix provided more nanocavities owing to the increased surface area and a thin residual layer among patterns compared to planar MIP system. This increased surface area contributed to the rapid transport of atrazine in MIP films and this leads to faster sensing responses. To evaluate the selectivity, herbicides analogous in structure to atrazine were used and the sensing behaviors were found almost identical with that in each herbicide analogues because of the limited non-specific chemisorption regardless of concentration. Once adding trace amounts of atrazine into the mixture solution, the sensor response showed 94% recovery of the original sensing response appeared in atrazine solution. Therefore, the lithographic approach to MIP sensing system provides rapid and ultra-sensitive detection in a selective way [29].

Voicu et al. [30] reported two dimensional (2D) molecular imprinting technique based on nano-templating and soft lithography techniques. In the basis of 2D molecular imprinting, nano-templating through microcontact imprinting was used. In the first step, by the attachment of a monolayer of the template, the stamp was modified. Then, the modified stamp was brought into contact with the monomer which provides the recognition groups that will interact with the template and then with the anchoring monomer which offers groups able to anchor the whole assembly to the substrate. In the next step, the stamp carrying the recognition and anchoring monomers on top was pressed against the substrate and the polymerization was initiated. In the study, the authors used theophylline as a model template. Several cycles of binding/extraction/rebinding of the template showed the stability of the MIP. Caffeine was used in testing the selectivity of the 2D MIP since caffeine has a very similar structure to theophylline. The results revealed that, even if both caffeine and theophylline were present in the same sample, rebinding of theophylline to the MIP was preferential and demonstrated selectivity towards theophylline. The methodology incorporated the advantages of oriented immobilization of the template and the attachment of the MIP on a substrate surface. By this way, homogeneous recognition sites were obtained on the surface. This system is promising for multi analyte detection at the same time by the immobilization of multiple molecular templates.

Dickert and Hayden [31] used soft lithography to prepare imprinted polymers for selective detection of yeast cells. The yeast imprinted QCM sensor surface showed a regular honeycomb like surface. The authors were able to monitor cell concentrations in the range between 10^4^ and 10^9^ cells/mL. The technique enabled the authors to generate highly regular imprints by microorganisms. Artificial recognition of microorganisms with MIPs depended on both chemical interactions and a large contact surface between yeast cells and polymer.

Jenik et al. [32] used the same technique to design a QCM sensor for two representatives of picornaviruses, human rhinovirus (HRV) and the foot-and-mouth disease virus (FMDV). In the study, it was shown that in contrast to natural antibodies, the entire surface of the virus particles was recognized by the MIPs because the differences in chemistry between the major and minor groups of the receptor sites which did not have any role in the detection. Weak non-covalent interactions were the main interactions in the recognition mechanism.

#### 2.2.2. Template Immobilization

**Figure 2:**
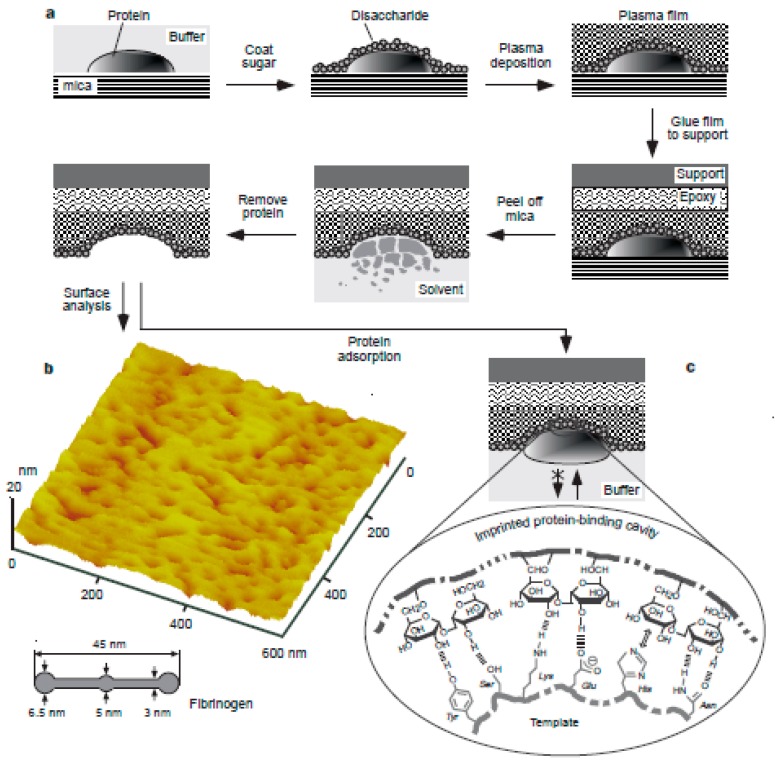
Protocol for template imprinting of proteins; (**a**) Template protein was adsorbed onto a mica and this surface was spin-coated with disaccharide to form a sugar overlayer. Plasma deposition was conducted to form a plasma film on top. The resulting plasma film was fixed to a glass cover slip. Then, mica was peeled-off and the protein was extracted. By this way, a nano-imprint with a shape complementary to the template protein was created on the imprint surface. (**b**) AFM image of the surface of a fibrinogen imprint, (**c**) Mechanism for the specific recognition of the imprinted surface. The main force for the selectivity was steric hindrance and an overall strong interactions which is due to many cooperative weak interactions including hydrogen bonds, Van der Waals forces and hydrophobic interactions (reproduced from [33] with permission).

Template immobilization approach was introduced by Shi et al. in 1999 [33]. In contrary to soft lithography in which template stamp is formed by using self-assembling, in this approach template molecule is immobilized on a solid support via chemical linkages [33,34]. Well-ordered imprinted surfaces can be produced by using this technique. In the general procedure of the technique, template protein is adsorbed onto a mica and a disaccharide layer is formed around the proteins. Then, radio-frequency glow-discharge plasma deposition is used to form polymeric thin films around the proteins coated with disaccharide molecules. The resulting plasma film is fixed to a glass support and mica is peeled-off in the next step. Then, the sample is soaked in a proper solution for dissolution and extraction of the template protein. By this way, nanocavities are created on the surface of the imprint with a shape complementary to that of the protein as shown in Figure 2 [33]. The disaccharides are covalently attached to the polymer film and they create polysaccharide like cavities which exhibit high selectivity for a variety of proteins including albumin, immunoglobulin G, lysozyme, ribonuclease and streptavidin [33,35,36,37].

Yılmaz et al. introduced “the use of immobilized templates” as a new approach in 2000 [34]. This procedure involves use of a silica based solid support to which the template is immobilized. It provides several advantages to these systems. For the insoluble templates in the polymerization cocktail, this approach might be used as a good alternative. The aggregation of templates in pre-polymerization mixtures which might be seen in some occasions can be prevented by this approach. In contrast to classical MIPs, no porogen was used for the imprinting of the immobilized template molecule in this technique. The pore structure of the obtained polymers was generated by dissolving the silica gel backbone. Because of this, all imprinted sites were located at or close to the surface of the pores which is an important advantage in facilitating the diffusion of the analyte to the binding sites.

Shiomi et al. used covalently immobilized hemoglobin (Hb) to create Hb-specific recognition cavities on silica. Covalent immobilization was achieved by forming imine bonds between amino groups on the protein surface and aldehyde groups on silica. After immobilization of the template, the organic silane monomers 3-aminopropyltrimethoxysilane (3-APTMS) and propyltrimethoxy-silane (PTMS) were polymerized on the Hb imprinted silica surface. Then, Hb was removed from the imprinted surface. The resulting protein imprinted silica showed good thermo-stability and mechanical strength [38]. Recently, several new variations of template immobilization including microcontact imprinting method have been introduced.

##### Microcontact Imprinting

Microcontact imprinting method was first described by Chou et al. in 2005 [39]. They prepared the microcontact imprints between two glass surfaces. In the general procedure of their method, template protein was immobilized onto the cover slip and this was treated with functional monomer to provide site-specific organization of the functional monomer by the template, thereby forming a complex between template and functional monomer(s). In the next step, a cross-linking monomer together with initiator were applied on the other glass and the micro-contact imprints were formed by bringing into contact with the cover slip that has the protein and monomer on top and the support glass carrying the cross-linker. Two functionalized surfaces were brought in contact under UV radiation. Then, the cover slip with the immobilized template molecules was removed from the surface and the excess polymer, non-reacted monomers and any leaking template molecule on the support were washed away. The quantification of re-binding was performed by using ELISA. The imprinted surfaces showed good selectivity for the template molecule (C-reactive protein, CRP) both in single binding and also in competition with human serum albumin (HSA). When the method is used to imprint HSA, the imprints demonstrated significantly greater affinity for the native template HSA than CRP this time. These promising results demonstrated the usability of the method to imprint relatively large proteins. Same authors used the same technique for several model antigens including lysozyme, ribonuclease A and myoglobin [40].

Microcontact imprinting technique allows for rapid and parallel synthesis of MIPs with different compositions [40,41]. Only a small amount of template is used to form a monolayer recognition surface that is an advantage for the templates which might be very expensive or available only in limited amounts. A few microliters of monomer solution are enough and dozens of samples can be polymerized at the same time using the same polymerization batch. Using this technique, potential solubility problems of macromolecular templates including proteins are also avoided. Since the template molecule is immobilized onto a glass support instead of being present in the monomer solution, the conformational stability of the template protein is preserved and aggregation of protein molecules is prevented [42].

Osman et al. [43] used microcontact imprinting technique to prepare SPR biosensor for myoglobin detection. They synthesized myoglobin imprinted poly (hydroxyethyl methacrylate-N-methacryloyl-L-tryptophan methyl ester) [poly (HEMA-MATrp)] nanofilm on the surface of a SPR sensor. The detection limit was found as around 88 ng/mL in the study. Functional monomer MATrp used in this study is monomer conjugated with amino acid which interacts with the functional groups on the template. Therefore, selective binding sites and structural memories for myoglobin are formed during the polymerization. In the selectivity studies, it was demonstrated that the imprinted nano-film recognized myoglobin preferentially over some competing proteins.

Lin et al. [40] reported the effect of cross-linking monomers for the microcontact imprinting of proteins. Four different types of cross-linking monomers: (ethyleneglycol dimethacrylate-EGDMA, tetraethyleneglycol dimethacrylate-TEGDMA, polyethyleneglycol 400 dimethacrylate-PEG400DMA, polyethyleneglycol 600 dimethacrylate-PEG600DMA) were tested and lysozyme rebinding on polyEGDMA, polyTEGDMA, polyPEG400DMA and polyPEG600DMA was measured by quantitative ELISA. The highest rebinding efficiency occurred on the lysozyme imprinted polyTEGDMA polymer. It was suggested that the size of the protein template may be correlated with the optimal number of EG-repeating units in the cross-linking monomers. More effective protein binding and imprinting was obtained with a cross-linking monomer containing more ethylene glycol repeating units. Longer cross-linkers are more flexible and this can lead to allow the polymeric matrix to ensure the protein’s conformational stability. The microcontact imprinting technique allows studies on interactions between the polymer surfaces and template molecules.

Sener et al. [42] developed a microcontact imprinted SPR biosensor for real-time detection of procalcitonin which is used as a biomarker to identify sepsis and to give an estimation of the severity of disease. The LOD value was calculated as approximately 3 ng/mL. The authors suggested that more sensitive detection may be possible by decreasing the polymer film thickness below 200 nm on the surface of the sensor chip. Selectivity results showed that the reflectivity change in sensor response in respect to the competing proteins human serum albumin (HSA), myoglobin and cytochrome c (cyt c) was almost the same for both the imprinted and non-imprinted SPR sensor. These results also indicate that the interactions between the imprinted surface and the competing proteins occur in a non-specific manner. Authors also tested the applicability of the sensor for procalcitonin (PCT) detection from simulated blood plasma which comprises HSA, immunoglobulins and fibrinogen at their typical concentrations in human blood plasma. When a comparison was made between the results obtained from analyzing the PCT samples with SPR and ELISA, it was shown that the agreement between two methods was approximately 99%.

The first microcontact imprinting study for the detection of a model analyte, BSA, with the capacitive biosensor was reported by Ertürk et al. [44]. In the study, a capacitive biosensor with an automated flow injection system was used for the detection of the analyte. In the detection principle of capacitive biosensors, the change in dielectric properties on the sensor surface when an analyte interacts with the biorecognition element is measured [45,46]. This measurement is very sensitive and selective, carried out in real-time and directly without using any labeling reagents. In the reported study, the authors prepared microcontact-BSA imprinted capacitive gold electrodes using MAA and PEGDMA as the functional monomer and cross-linker, respectively. After optimization of the conditions, BSA detection was carried out within the concentration range of 1.0 × 10^−20^–1.0 × 10^−8^ M with a LOD value of 1.0 × 10^−19^ M. The results showed that the BSA imprinted electrodes were selective for BSA compared to HSA and IgG. The cross-reactivity ratios were 5% for HSA and 3% for IgG. A total of 80 assays during a period of 2 months were carried out with the same electrode without any significant differences in the detection performance. The developed method was promising with the advantages of high sensitivity, selectivity and operational stability.

**Figure 3:**
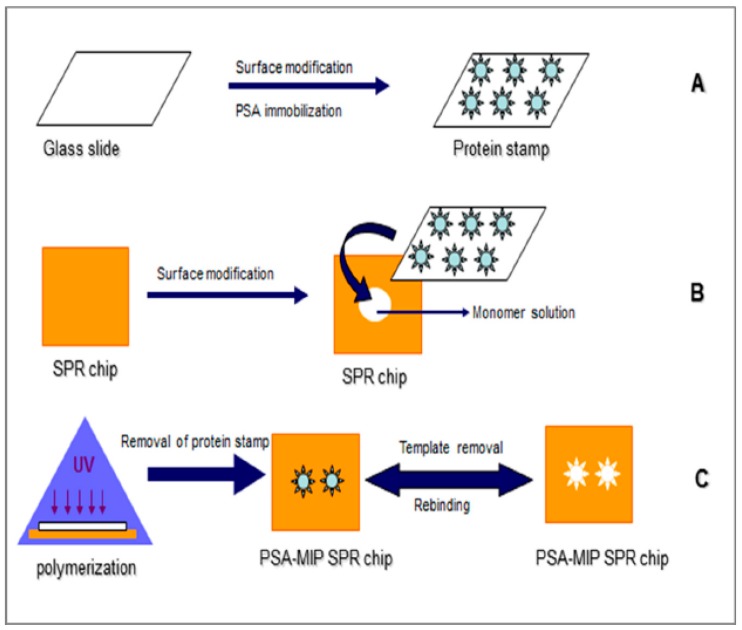
Schematic representation of microcontact imprinting of PSA onto the SPR biosensor surface: (**A**) Preparation of glass cover slips (protein stamps); (**B**) preparation of SPR chips; (**C**) microcontact imprinting of PSA onto the SPR biosensor chip surface via UV-polymerization (reproduced from [52] with permission).

In their next study, the same authors used the same technology for ultra-sensitive detection of an important biomarker, prostate specific antigen (PSA) for the early diagnosis of prostate cancer. A microcontact-PSA imprinted SPR sensor chip was prepared as shown schematically in Figure 3 and PSA detection was performed with standard PSA solutions in the concentration range of 0.1–50 ng/mL with a LOD value of 91 pg/mL. The LOD value obtained from the study was comparable to the LOD values reported in previous studies [47,48,49,50]. Because the cut-off value between normal and possible pathological levels of PSA in human serum is 4.0 ng/mL, the developed system with this LOD value meets the requirements of PSA analysis from clinical samples. In the final step, PSA detection was also carried out from prostate cancer patients’ serum samples and the results were compared with the ELISA results. The agreement between two methods, SPR and ELISA, was found as approximately 98% which established the reliability and the sensitivity of the method for PSA detection. All the results revealed that the system could be considered as a promising tool and used as an alternative to ELISA for PSA detection in standard PSA solutions and clinical samples. When the authors compared the sensitivity from analysis done with a SPR system using microcontact-PSA imprinted sensor surface with results from a capacitive system with microcontact-PSA imprinted electrodes, the capacitive system was approximately 1000 times more sensitive than the SPR system [51]. Capacitive biosensor technology with the microcontact imprinting method might be used successfully for real-time detection of various analytes even in very low concentrations [52]. Microcontact imprinting has also been used for developing assays of whole cells. Idil et al. [53] monitored *E. coli* cells using a capacitive measuring approach. The sensor chip was essentially prepared according to the procedures used for sensor chips against proteins. The assay was selective against the imprinting microorganism, but there was still some cross-reactivity against competing bacteria. A limit of detection was 70 CFU/mL. The approach on assaying cells via exploitation of microcontact imprinting is promising, but there is still room for improvements [53].

#### 2.2.3. Surface Imprinting via Grafting

In surface grafting, template molecule is adsorbed or attached with the polymeric functional groups which are already grafted on the surface of the support. In other words, contrary to template immobilization method, template molecule is present during the polymerization step in this method [9]. The advantages of the method are improved affinity interactions because of faster mass transfer as a result of higher analyte mobility, better control over polymer shape and morphology.

A molecularly imprinted polymer for domoic acid (DA) was synthesized by Lotierzo et al. [54] by direct photo-grafting onto the SPR gold chip surface. Self-assembly of 2-mercaptoethylamine (2-MEA) was used for the surface functionalization of the SPR gold chip. Then, carbodiimide chemistry was performed for the covalent attachment of the photo initiator to the surface. By using a photo-initiator with symmetrical carboxylic acid group at each arm, covalent attachment of the initiator to the amino-functionalized gold surface was possible by using carbodiimide chemistry. 2-(diethylamino)ethylmethacrylate and EGDMA were used as functional monomer and cross-linker, respectively and thin polymeric film formed only on the surface. The measured MIP film thickness was 40 nm since the immobilization of the photo-initiator to the gold surface prior to being treated with pre-polymerization mixture resulted in the polymerization reaction took place to the close vicinity of the gold surface. The developed system had approximately three times higher detection limit compared to that of monoclonal antibody immobilized system.

BSA surface-imprinted thermosensitive magnetic composite microspheres were prepared via surface grafting co-polymerization method. Temperature sensitive N-isopropylacrylamide (NIPAm) and the functional monomer methacrylic acid (MAA) were used as co-monomers and methylene bis-acrylamide (MBA) as the cross-linking agent. The adsorption-desorption of template molecule was regulated by changing the system temperature due to the thermo-sensitive imprinting layer [55].

**Figure 4:**
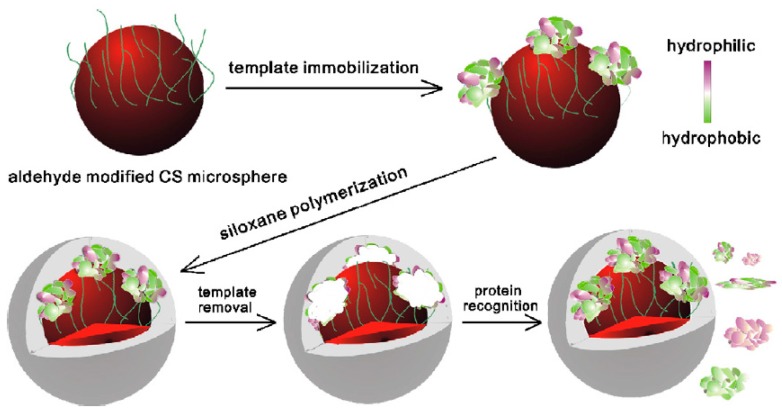
Schematic representation of synthesis of protein imprinted polymers on CS microsphere using immobilized protein as a template. The synthesis involved three steps; Firstly, template BSA was covalently immobilized on the polysaccharide core by forming imine bonds. The second step involved siloxanes polymerization on the polysaccharide–protein surface. It resulted in a polymeric network molded around the template. The template protein was removed in the third step. Cavities complementary to the template protein in shape, size, and functional group orientation were created (reproduced from [56] with permission).

An interfacial organic-inorganic hybridization concept was used for the preparation of the spherical imprinted materials. In this surface imprinting study, model template BSA was covalently immobilized by forming peptide bonds with the functional amine groups of biopolymer chitosan [56]. Then, two different kinds of organic siloxanes 3-aminopropyltrimethoxysiloxane (3-APTMS) and tetraethoxysiloxane (TEOS) were assembled. In the next step, polymerization was performed on the polysaccharide-protein surface via sol-gel process. In the last step, template protein BSA was removed from the surface and cavities complementary to the template in size, shape and orientation of the functional groups were created on the surface as shown schematically in Figure 4. Cytochrome c, transferrin, beta-amylase and lysozyme were used as competing proteins. Compared to the imprinted material, the control, non-imprinted material showed poor adsorption performance. The grafting of the imprinted layer through interfacial organic-inorganic hybridization improved stability and reproducibility properties of the final material.

**Figure 5:**
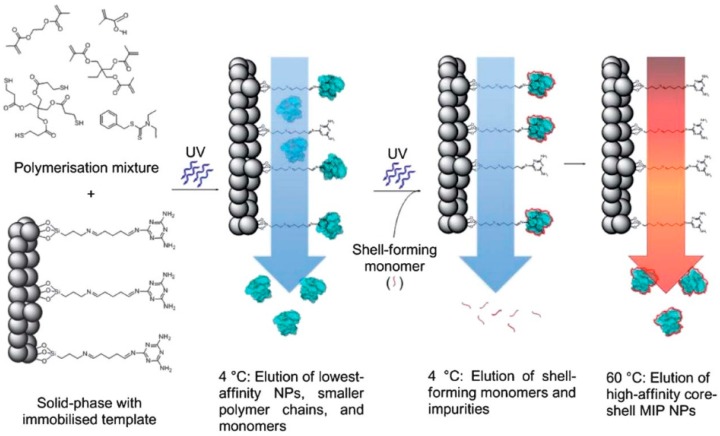
Schematic representation of core-shell MIP NPs. The template molecule was immobilized onto a solid support. A polymerization mixture (containing functional and cross-linking monomers, initiators) were loaded on this solid support. Polymerization was initiated via UV light. Un-reacted monomers and low-affinity particles were eluted from the solid phase at low temperature where the high affinity particles remained bound to the template. The high-affinity particles were subsequently grafted with a secondary monomer and an application of UV-light. After polymerization, high-affinity core-shell MIP NPs were eluted from the shell by increasing the temperature up to 60 °C (reproduced from [58] with permission).

The first report of the automated synthesis of imprinted polymer nanoparticles (nanoMIPs) with size, specificity and solubility characteristics for industrial manufacturing was published by Poma et al. [57]. The protocol developed for the automated synthesis and purification of MIP nanoparticles (Figure 5) was as follows [58]:

(1) In the first step, monomer/initiator mixture was dissolved in an appropriate solvent and then loaded onto a temperature controlled column reactor. This column reactor consisted of the template immobilized onto a solid support.
(2) Then, in the next step, when the optimum reaction conditions were obtained, polymerization was initiated by UV-irradiation of the reactor.
(3) After polymerization, the column reactor was washed with a solvent which resulted in the elution of unreacted monomers and other low molecular weight materials together with low-affinity polymer nanoparticles. For the other high-affinity bound particles, collection was managed by increasing the column temperature or by addition of auxiliary reagents like formic acid.

The advantages of this approach are: uniform binding properties, elimination of contamination of the product with template because immobilized templates are used, re-usability of the template in further applications, ease of the procedure, obtaining pure final product which eliminates the need for post-purification steps. Poma and co-workers indicated that by using this technique, high-quality MIP nanoparticles similar to monoclonal antibodies can be produced and these MIP nanoparticles offer a lot of advantages over antibodies for use in assays and sensors in the future.

#### 2.2.4. Emulsion Polymerization

Another surface imprinting strategy is emulsion polymerization. In the general procedure of the technique, core particles are synthesized before they are covered with imprinted shells.

Tan et al. [59] used surface imprinting strategy based on covalently immobilized templates to prepare BSA-imprinted sub-micrometer particles via two-stage core shell mini-emulsion polymerization. Through the encapsulation of Fe_3_O_4_ nanoparticles, the MIP particles gained magnetic properties which increases their potential applications in various fields including magnetic bioseparation, cell labeling and bioimaging. Imprinted particles with non-immobilized (free) template molecules (fMIP) did not display the expected template recognition and this illustrates the importance of the template immobilization for a successful surface imprinting. The MIP prepared according to this strategy displayed high specific recognition for BSA without any adsorption of the competitive proteins. Therefore the technique has potential to be used for the preparation of highly selective biosensor surfaces in further applications.

**Figure 6:**
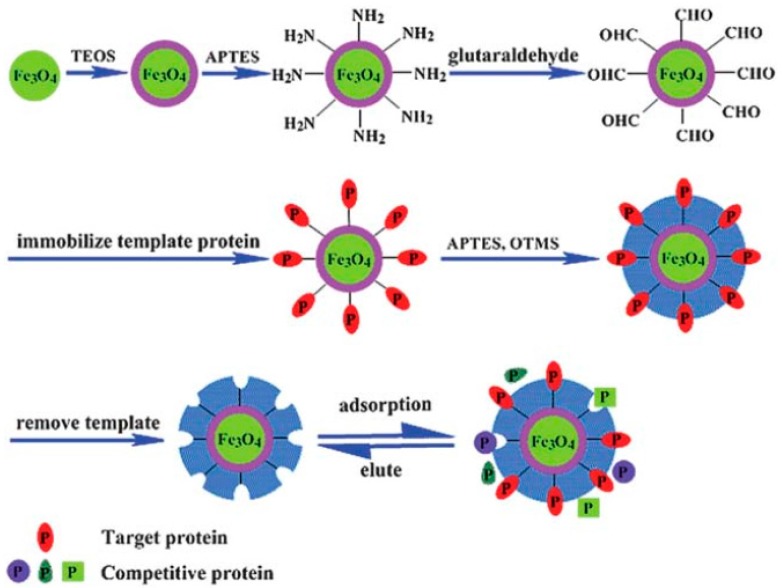
General method for synthesis of molecularly imprinted magnetic Fe_3_O_4_@MIP NPs using an immobilized template protein. In the protocol, the surface of Fe_3_O_4_ NPs was transformed to a silica shell using tetraethoxysilane (TEOS) to form Fe_3_O_4_@SiO_2_. Then, the surface of Fe_3_O_4_@SiO_2_ was reacted with 3-aminopropyltriethoxysilane (APTES) to introduce amino groups on the surface which were reacted with glutaraldehyde (GA) in the following step to form aldehyde modified Fe_3_O_4_@SiO_2_ NPs. In the next step, template protein was covalently immobilized on the aldehyde modified Fe_3_O_4_@SiO_2_ NPs. In the last step, siloxane co-polymerization by APTES and octyltrimethoxysilane resulted in a polymeric network around the template protein on the surface of the Fe_3_O_4_@SiO_2_ – protein complex. Then, the template was removed from the surface and imprinted cavities which were complementary to the template in size, shape and functional groups were obtained (reproduced from [60] with permission).

Thin MIP coatings on magnetic Fe_3_O_4_ nanoparticles (NPs) with a uniform core-shell structure were developed in a study [60] (Figure 6). BSA, bovine hemoglobin, RNase and lysozyme were used as the templates. A combination of surface imprinting and sol-gel techniques was used for synthesizing the magnetic protein selective MIPs. Bovine hemoglobin imprinted Fe_3_O_4_ NPs showed the best template recognition and highest adsorption capability compared to other MIPs. To determine the applicability of Fe_3_O_4_ for binding bovine hemoglobin-MIPs from complex real samples, selective capture of bovine hemoglobin from bovine blood samples was tested. It was shown that, BHb was almost completely removed from the blood sample after treatment with BHb-MIPs which suggests the potential of MIPs in practical applications including the recognition and enrichment of proteins. Because of the great potential of the technique to capture the target analyte almost completely from the blood sample, the technique is promising to develop biosensors especially for selective detection of analytes which are found in complex real samples.

The tendency of bacteria to assemble at oil-water interfaces was used in a study to create bacteria-imprinted recognition sites on the surface of polymer beads. The authors demonstrated that MIPs for bacterial recognition can be prepared from bacteria based pre-polymer networks which are also known as Pickering emulsions [61]. The basic principle of the technique is the partitioning of solid particles between two immiscible liquids. In this study, living microorganisms were used as particle stabilizers in Pickering emulsion. The general procedure of the technique involves three steps:

(1) In the first step, negatively charged bacteria were assembled with a positively charged pre-polymer which contained vinyl groups,
(2) In the next step, bacteria-pre polymer complexes were used as the particle stabilizer to form emulsion of a cross-linking monomer (the oil phase) in water. The cross-linking monomer was polymerized by free radical polymerization and by this way the pre-polymer was covalently fixed to the core of the polymer beads.
(3) After template removal, bacteria-imprinted sites were obtained on the surface of the polymer beads.

N-acrylylchitosan (NAC) was used as the vinyl-containing pre-polymer. The binding experiments of the groups of bacteria proved that the bacterial recognition on the imprinted polymer beads was dependent on the nature of the polymers and the target bacteria.

Micro-emulsion polymerization was used to construct glutathione peroxidase-like active sites on polystyrene nanoparticles [62]. Two functional monomers were used in the model system. One of them was introduced on the surface of the nanoparticle and acted as a catalytic center while the other was designed as a binding site for the complexation of the carboxyl group of the substrate. The imprinted polymer showed very high catalytic activity and substrate specificity. When the advantages of the glutathione peroxidase mimics including high catalytic activity, good water solubility and substrate specificity are taken into consideration, these imprints might have potential applications in medicine.

### 2.3. Epitope Imprinting

Rachkov and Minoura [63] proposed an alternative method to the use of whole molecule as templates. In their method, a domain with the same sequence as one of the terminal chains of the target protein was used as template. In this “epitope approach”, the peptide epitope was covalently attached to a glass/silicon surface on which the monomers were polymerized in the next step. By this way, after removal of glass/silicon with the target molecules, a molecularly imprinted polymer (MIP) film was produced. In the following steps, the MIP was used to bind and capture the target protein from protein mixtures.

In the epitope imprinting approach, more specific and stronger interactions can be obtained by using a small part or fragment of a macromolecule. Therefore, non-specific binding can be minimized and the affinity can be improved. The imprinted polymer has the ability to recognize the template as well as the entire protein when the sequence is exposed.

“C termini” of proteins are selected as “epitopes” for the imprinting process because these sites are generally less frequently prone to post-translational modifications and the minimum length of peptide necessary to create “unique” recognition for the whole target protein has been reported to be around 9 amino acids according to Nishino et al. [64]. The short epitopes target the primary structure of the peptide rather than the more complex secondary and tertiary structures. Therefore, this approach is also promising for the capture of target proteins based only on genomic information.

Tai et al. [65] used a 15-mer peptide, which is a linear epitope of the dengue virus NS1 protein, as a template to prepare molecularly imprinted QCM sensors. The polymers imprinted with a peptide recognized both the template and other proteins which possess the same epitope part in the structure. The system is ideal as an in vitro cellular assay for quantitatively recognizing proteins.

Ertürk et al. [6] developed an immunoglobulin G sensor via imprinting the F_ab_ fragments of the antibody onto a SPR sensor chip surface. F_ab_ fragments are smaller than the IgG molecule and they are the functional components of the whole molecule. The developed system had showed high affinity to the IgG molecule and the system was also suitable to detect IgG from human plasma with approximately 99% precision in the studied concentration range. Correlation between the developed method and ELISA was found to be approximately 99%. The selectivity coefficients showed that the F_ab_ imprinted sensor’s selectivity for IgG was 21 and 14 times higher than that of BSA and F_c_ fragments, respectively. By using the epitope imprinting approach in the study, it was aimed to overcome some of the drawbacks of macromolecule imprinting. The results revealed that the sensor could detect IgG in a highly sensitive and specific way even from human plasma with a high agreement with results from conventional ELISA method.

**Figure 7:**
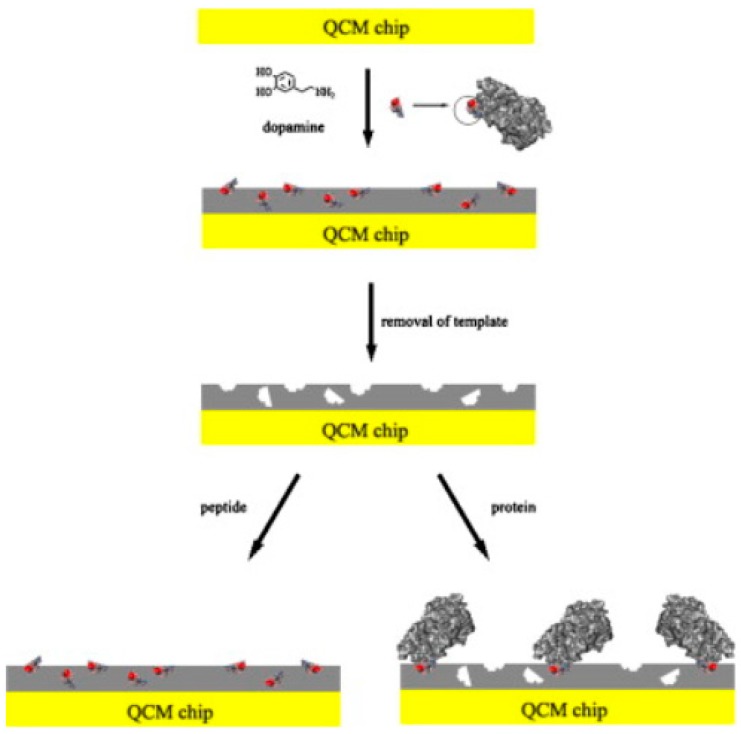
Schematic diagram of epitope imprinting. Dopamine was used as functional monomer and cross-linker. The synthetic peptide was embedded in the polydopamine film on the QCM sensor chip. After template removal step, the recognition sites which were complementary to the template peptide and able to recognize the whole protein were formed on the surface (reproduced from [66] with permission).

Gp41 (HIV type-1 related glycoprotein 41) is a transmembrane protein which plays an important role in the early diagnosis of immunodeficiency syndrome (AIDS). Therefore, ultra-sensitive detection of this protein is especially important in early diagnosis and monitoring of pathogenic conditions. Lu et al. [66] developed a biomimetic sensor for the detection of gp41 using epitope imprinting approach. The authors used dopamine as functional monomer and cross-linking agent as shown in Figure 7. The advantages of using dopamine were high hydrophobicity and biocompatibility of the prepared sensor surface. During the polymerization of dopamine on QCM sensor chip, the template peptide was embedded in it. It was indicated that the thickness of poly-dopamine film would affect the distribution of the imprinted sites as well as the rebinding capacity. Therefore the authors demonstrated that when 5 mg/mL dopamine was used for the polymerization, the MIP coated QCM chip reached maximum template binding capacity. A thicker film would lead into a decrease in rebinding which would mean lower sensitivity. The LOD value for HIV-1 gp41 protein was reported as 2 ng/mL. When the sensor was used for the detection of the protein in human urine sample, the recovery was found in the range of 86.5%–94.1%.

## 3. Concluding Remarks

The area of molecular imprinting is in a rapid development phase at present. What earlier was regarded as impossible is now certainly reachable with new technology. This is illustrated in the present review, by e.g., imprinting of proteins and microbial cells.

The development of nanoparticles with imprinted structures opens new possibilities both in analysis and in the medical area. Synthesis of bi-functional MIPs may be a reality in the future, and then the field is open for e.g., sandwich assays.

As the MIPs are now approaching the sizes of immunoglobulins one can imagine that stable MIPs may replace antibodies in many applications in harsh environments and when repeated assays are to be performed. It also opens up for more frequent on-line assays based on binding reactions since then exchange of the affinity binder is far less frequent. There is still a lack of good information on molecular properties of MIPs and then also on means to improve stability features of structures that are regarded as already very stable.
